# Biodegradable and Insoluble Cellulose Photonic Crystals and Metasurfaces

**DOI:** 10.1021/acsnano.0c03224

**Published:** 2020-06-19

**Authors:** Vincenzo Caligiuri, Giacomo Tedeschi, Milan Palei, Mario Miscuglio, Beatriz Martin-Garcia, Susana Guzman-Puyol, Mehdi Keshavarz Hedayati, Anders Kristensen, Athanassia Athanassiou, Roberto Cingolani, Volker J. Sorger, Marco Salerno, Francesco Bonaccorso, Roman Krahne, José Alejandro Heredia-Guerrero

**Affiliations:** † Istituto Italiano di Tecnologia, Via Morego 30, 16163 Genova, Italy; ‡ Dipartimento di Fisica, Università della Calabria, 87036 Rende, Italy; § CNR Nanotec, Università della Calabria, 87036 Rende, Italy; ⊥ CIC nanoGUNE, Tolosa Hiribidea 76, 20018 Donostia-San Sebastian, Basque Country, Spain; ◊ Department of Electrical Engineering, University of Notre Dame, Notre Dame, Indiana 46556, United States; ∥ IHSM La Mayora, Departamento de Mejora Genética y Biotecnología, Consejo Superior de Investigaciones Científicas, E-29750 Algarrobo-Costa, Málaga, Spain; △ Department of Electrical and Computer Engineering, George Washington University, Washington, DC 20052, United States; ¶ Department of Engineering, Durham University, Durham DH1 3LE, United Kingdom; □ Department of Health Technology, Technical University of Denmark, DK-2800 Kongens Lyngby, Denmark; ▽ Materials Characterization Facility, Istituto Italiano di Tecnologia, Via Morego 30, 16163 Genova, Italy; ▼ BeDimensional Srl., Via Albisola 121, 16163 Genova, Italy

**Keywords:** cellulose, cocoa agro-waste, biodegradability, water insolubility photonic crystals, meta-structures, plasmonic colors, SERS

## Abstract

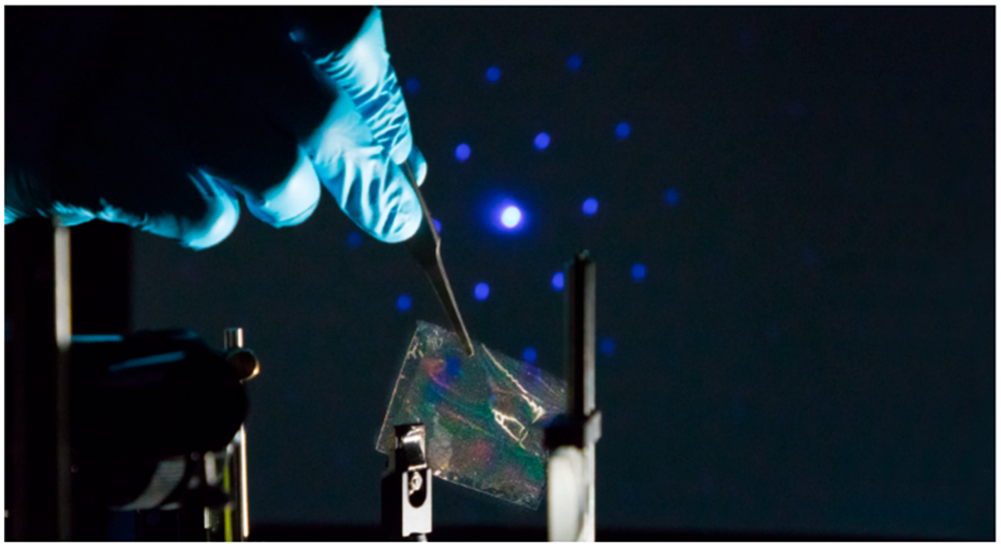

The replacement of plastic with eco-friendly and biodegradable materials is one of the most stringent environmental challenges. In this respect, cellulose stands out as a biodegradable polymer. However, a significant challenge is to obtain biodegradable materials for high-end photonics that are robust in humid environments. Here, we demonstrate the fabrication of high-quality micro- and nanoscale photonic and plasmonic structures *via* replica molding using pure cellulose and a blended version with nonedible agro-wastes. Both materials are biodegradable in soil and seawater according to the ISO 17556 standard. The pure cellulose films are transparent in the vis–NIR spectrum, having a refractive index similar to glass. The microstructured photonic crystals show high-quality diffractive properties that are maintained under extended exposure to water. Nanostructuring the cellulose transforms it to a biodegradable metasurface manifesting bright structural colors. A subsequent deposition of Ag endowed the metasurface with plasmonic properties used to produce plasmonic colors and for surface-enhanced Raman scattering.

Nowadays, the demand and production of photonic structures is rapidly growing, and large surface integration capabilities are often required. In this respect, the fabrication technique of choice at both the micro- and nanoscale is *replica molding*. Its advantages are numerous, from the straightforward and nondemanding implementation to the possibility of a fast and low-cost production over large surfaces.^1−6^ However, the polymers typically involved in replica molding (*e*.*g*., PDMS, polyurethanes, PMMA) are not biodegradable and their demand is steadily rising, making it an environmental and possibly a health-safety concern.^7−9^ This is because the product life-cycle (from stocking, over use, to recycling) bears a plurality of potentially harmful factors for both health and environment such as the use of fossil fuels and their chemical conversion in the production process, the presence of chemical additives such as bisphenol A (BPA), and, last but not least, the non-biodegradability. Therefore, numerous biodegradable alternatives to common plastic polymers have been proposed.^10−17^ In the framework of photonic technologies, structures inspired by nature have been widely analyzed as biocompatible platforms;^18−23^ for instance, encouraging results in producing biocompatible and more eco-friendly polymers suitable for replica molding have been realized with silk and silk derivatives.^24−30^ Another promising material is cellulose;^31^ given its high availability with an estimated annual world biomass production between 10^11^ and 10^12^ tons per year,^32^ and because of the consolidated industrial process procedures of extraction, isolation, and purification, cellulose already demonstrated valuable functionality such as a biodegradable, sustainable polymer in food packaging, textiles, building industry, drug delivery, foldable electronics, and additive manufacturing.^16,33−39^ Recently, cellulose derivatives (*e*.*g*., hydroxypropyl cellulose and sulfonated cellulose) demonstrated promising results in the replica of photonic structures.^40−44^ Nevertheless, in order to improve the solubility and processability of these materials, chemical modification of pure cellulose is necessary.^40^ Moreover, the high hygroscopy and water solubility of the resulting derivatives make photonic structures from these materials almost incompatible with humid and liquid environments.^45^ In this work, we demonstrate the possibility of replicating microscale photonic structures and nanoscale metasurfaces by using pure cellulose as a replica-molding polymer. The cellulose films have a refractive index and transparency that is well-suited for photonic structures with an operating range in the visible–near-infrared (vis–NIR) spectrum, which is paired with excellent replication in terms of accuracy and large surface patterning. Furthermore, the obtained films are insoluble in water, which significantly enlarges their application range, for example in plasmonics and in photonic sensing, where humid and liquid environments are common.^46^ At the same time, they are fully biodegradable in soil and in seawater, according to the ISO 17556 standard. Here, we demonstrate the successful structuring of the cellulose and cellulose/agro-waste blended films with large-area (∼16 cm^2^) hexagonal and square lattice arrays with micro- and nanoscale periodicity. The microscale gratings lead to high-quality diffraction patterns with good diffraction efficiency performances, and the nanostructured metasurfaces result in structural dielectric and plasmonic design-tunable colors. In the latter case, metal-coated cellulose films were also tested as surface-enhanced Raman scattering substrates. In this case, the plasmonic nature of the metasurface has been exploited to significantly enhance the Raman performances when the excitation beam is tuned to the plasmonic resonance. Our replica-molding methodology can be applied also to nonedible, inexpensive, and underutilized agro-wastes such as cocoa pod husks, which improves the already outstanding biodegradability of the pure cellulose while preserving the optical performance.

## Results and Discussion

**Figure 1:**
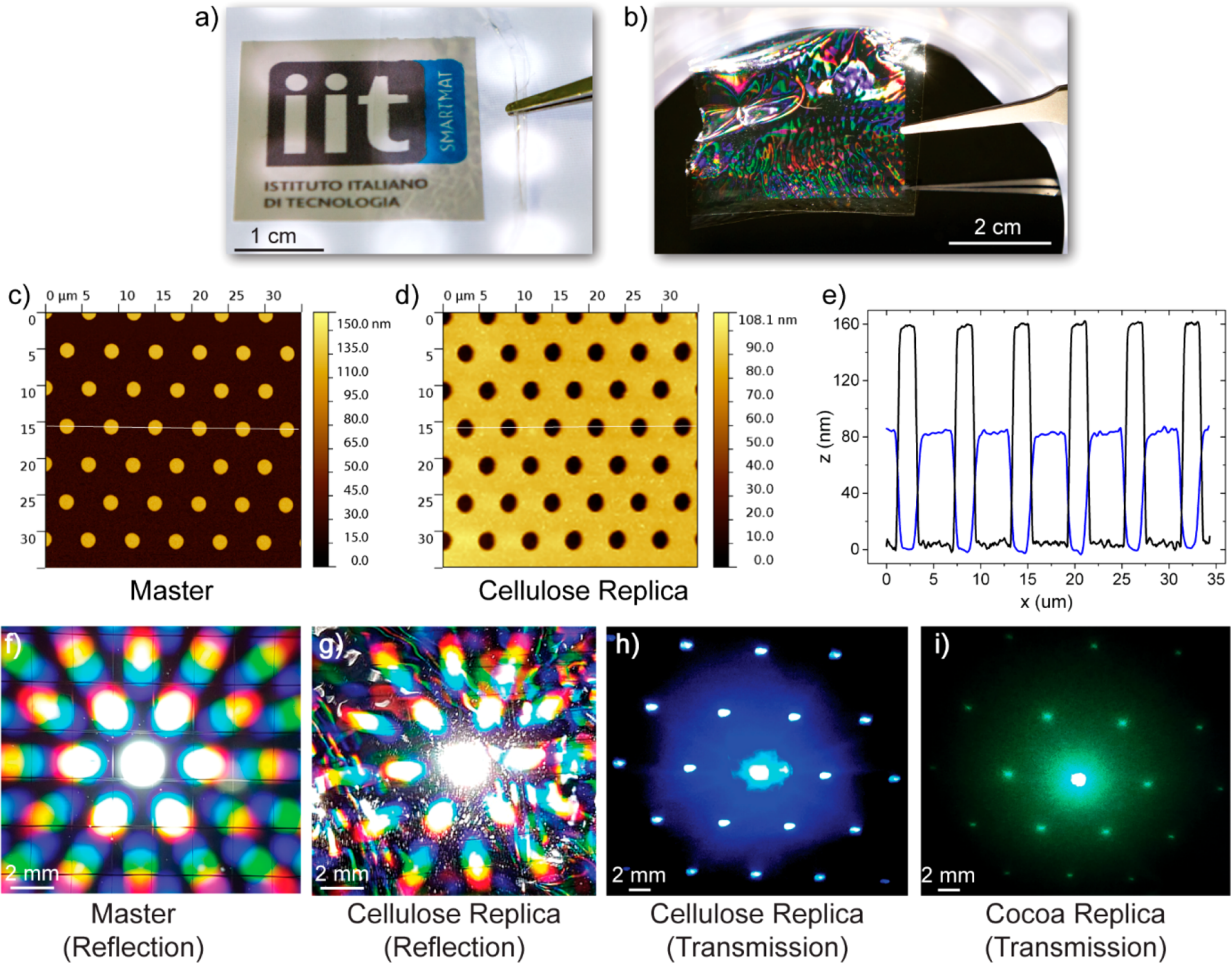
Transparency, replica precision, and optical diffraction of micropatterns. (a) Photograph of a free-standing cellulose film that demonstrates its excellent transparency by partially covering the IIT logo. (b) Micropatterned cellulose film with 4 cm × 4 cm dimensions after it was peeled of the master. (c, d) AFM images of the master and the cellulose replica showing a hexagonal array of pillars and holes, respectively. The nominal pillar diameter is 2 μm, and the periodicity is 6 μm. (e) Height profiles of the master (black) and replica (blue) along the lines indicated in (c) and (d). (f, g) Diffraction patterns recorded in reflection from master and replica. (h, i) Diffraction patterns recorded in transmission from the cellulose (h) and cocoa (i) replicas under illumination with laser light at 405 and 510 nm wavelength, respectively. The use of the IIT logo reproduced in panel (a) has been courteously authorized by the Italian Institute of Technology.

**Scheme 1:**
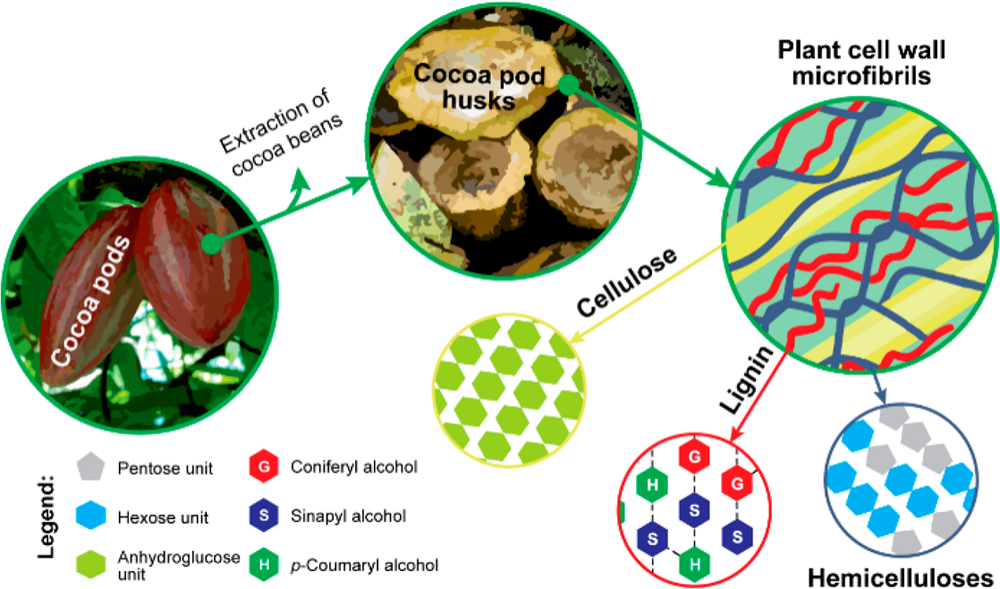
Different components of cocoa pods after the extraction of beans. The distribution and chemical composition of cellulose, lignin, and hemicellulose are indicated. Adapted with permission from Bayer, I. S.; Guzman-Puyol, S.; Heredia-Guerrero, J. A.; Ceseracciu, L.; Pignatelli, F.; Ruffilli, R.; Cingolani, R.; Athanassiou, A. Direct Transformation of Edible Vegetable Waste into Bioplastics. *Macromolecules***2014**, *47*, 5135–5143. Copyright (2014) American Chemical Society.

Pure cellulose solutions were prepared by solubilizing the polysaccharide in a mixture of trifluoroacetic acid (TFA) and trifluoroacetic anhydride (TFAA). The advantage of using TFA and TFAA as solvents is that they allow cosolubilizing organic matter from the cell walls of cocoa pod husks (*e*.*g*., hemicelluloses, lignin, fats, *etc*.) as illustrated in Scheme 1 in the Methods section, which leads to a highly biodegradable replica-molding materials.^47^ The cellulose films were produced by drop-casting the solution on suitable substrates and letting the solvent evaporate under ambient conditions. Figure 1a demonstrates that the free-standing cellulose film is fully transparent across the visible range (transmission around 90% from 400 to 900 nm; see Figure S1a of the Supporting Information), with a refractive index around 1.565 (see Figure S1b,c,d), which is close to that of glass and most common polymers in optical technology.^48,49^ For replica molding we used two different patterns: a hexagonal lattice with microscale dimensions and a cubic lattice with a periodicity of 400 nm. Following this process, we demonstrate a macroscale (4 cm × 4 cm) yet microscale-patterned cellulose film replica (Figure 1b). Here we used a hexagonal array of SiO_2_ pillars with a diameter of 2 μm and periodicity of 6 μm as master for the molding (see AFM image in Figure 1c) and obtained high-quality cellulose molds with a hexagonal pattern of microscale holes (Figure 1d). The AFM line profiles of master and replica confirm the accuracy of the pattern transfer to the cellulose replica. The high-quality morphological and optical properties of the cellulose molds are reflected in their diffraction patterns that resemble closely that of the master,^50−53^ as demonstrated in Figure 1f,g, in reflection. The diffraction patterns of the microstructured cellulose and cocoa films under illumination with blue (405 nm) and green (510 nm) laser light show well-defined hexagonal patterns. The very good diffractive properties have been confirmed in the analysis reported in Section 2 of the Supporting Information. Cocoa films obtained with only a minor portion of cellulose (10 wt %) show biodegradability performances in terms of mg O_2_/L about 8 times higher than pure cellulose, and, despite their lower transparency, they maintain high optical diffraction performances stemming from the accurate replication of the pattern (see Figure S2 of Section 2 for the diffraction efficiency measurements and Figure S3 of Section 3 of the Supporting Information for the SEM analysis showing the morphology of both the cellulose and the cocoa films).

### Biodegradability in Soil and Seawater and Insolubility

**Figure 2:**
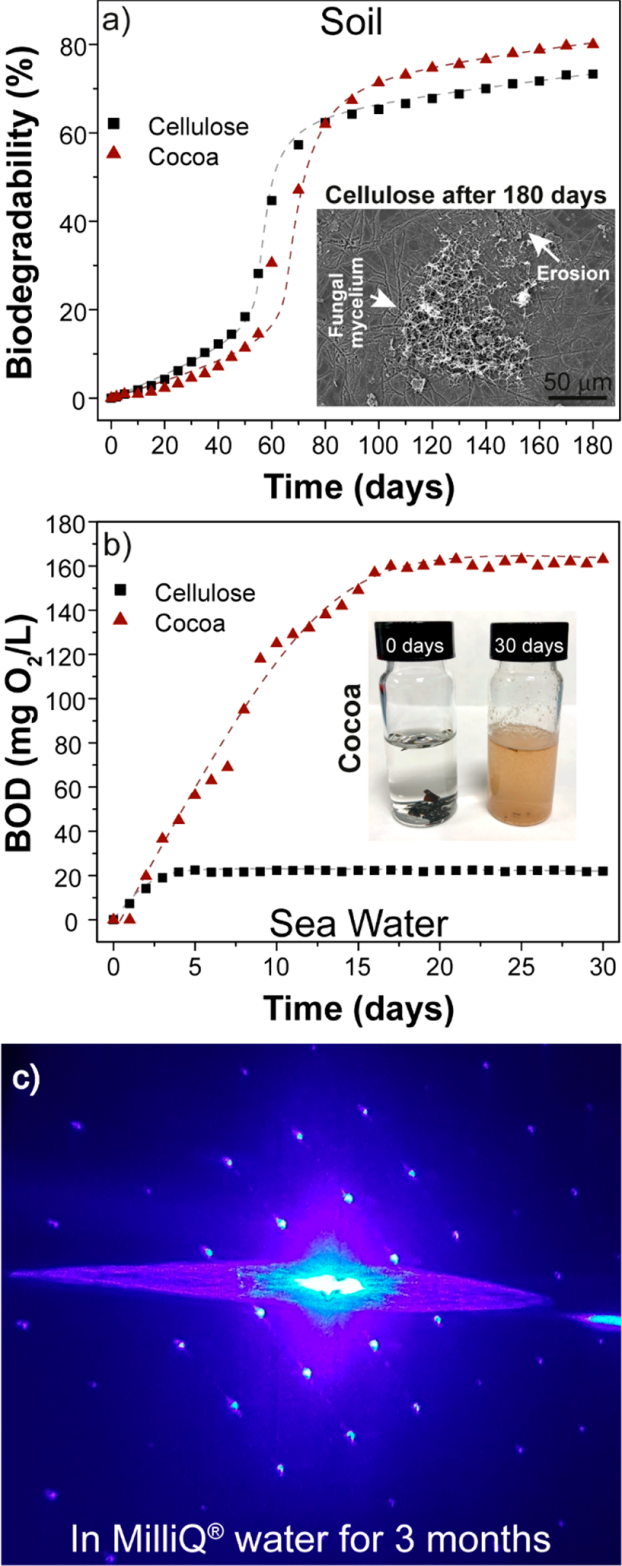
Biodegradability. (a) Biodegradability in soil of cellulose and cocoa pod husk materials. (b) BOD data in seawater of cellulose and cocoa pod husk materials. (c) Diffraction pattern of the hexagonal cellulose PC taken with the sample in ultrapure Milli-Q water for three months.

The strong point of the cellulose and mixed cellulose/cocoa blends that we obtain is their high biodegradability both in soil and in seawater, which translates into rather low solubility in purified and deionized water. This combination enables a truly biodegradable material platform for optical applications that operates in both a humid atmosphere and aqueous environments. Figure 2a shows the biodegradability of the cellulose and cocoa films in soil over a period of six months. Both materials exhibited a sigmoidal biodegradation trend as a function of time, with three different slopes: (i) an initially low degradation followed by (ii) an increase, resulting in an intermediate regime of accelerated degradation with a significantly steeper slope (*high-increase* zone), and (iii) a final saturation of the rate increase leading to a plateau. Cellulose shows higher percentages of biodegradation in the first two regimes and reaches the high-increase and the plateau zone earlier than the cocoa pod husks blend. However, in the final regime, the biodegradability of the cocoa pod husk material (∼80%) is higher than for cellulose (∼73%). Both materials can be considered biodegradable in soil according to the ISO 17556 standard, which requires biodegradation of 60% of the material after half a year. In fact, the growth of fungi and the erosion produced during the biodegradation process was clearly observed by SEM, as can be seen in the inset of Figure 2a. The biodegradability was also tested in seawater by measuring the biochemical oxygen demand at 30 days (BOD30), Figure 2b. For the pure cellulose films, the biodegradation started after 1 day and reached a plateau after 5 days at 22.5 mg O_2_/L. In the case of cocoa pod husks, the degradation started after 2 days, reaching a maximum oxygen consumption after 17 days at 160 mg O_2_/L. The higher biodegradability of the cocoa pod husks as compared to cellulose can be related to the presence of hemicelluloses, pectin, sugars, proteins, and fats in the agro-waste that microorganisms can metabolize to obtain energy.^54,55^ In contrast, polymers that are typically used in replica molding such as poly(methyl methacrylate) (PMMA), polyurethanes, and polydimethylsiloxane (PDMS) are non-biodegradable; for instance, PMMA displays rather low decomposition rates when different fungi, soil invertebrates, and microbial communities were used to biodegrade it.^8^ The biodegradability of polyurethane depends strongly on its chemical composition; however, it can be considered resistant to microorganisms.^7^ PDMS is practically not biodegradable in seawater due to its low surface energy and intrinsic hydrophobicity, while its half-life in soil ranges between 2.4 and 3.9 years.^56^ We demonstrate the insolubility of our microstructured cellulose films that act as photonic crystals by recording its diffraction pattern in water over time. Figure 2c shows the diffraction pattern recorded after immersion in distilled water for 3 months, which closely resembles that acquired in free space shown in Figure 1h and, therefore, highlights the excellent water solubility resistance. The persistence of the diffraction pattern over such a long immersion in water demonstrates the humidity-independent optical response of the cellulose structures. This constitutes a significant advantage with respect to some biodegradable yet insoluble polymers that still suffer from swelling/contraction of their morphology when exposed to a humid environment.^57−61^

### Dielectric and Plasmonic Colors and Surface-Enhanced Raman Scattering (SERS) Effect

**Figure 3:**
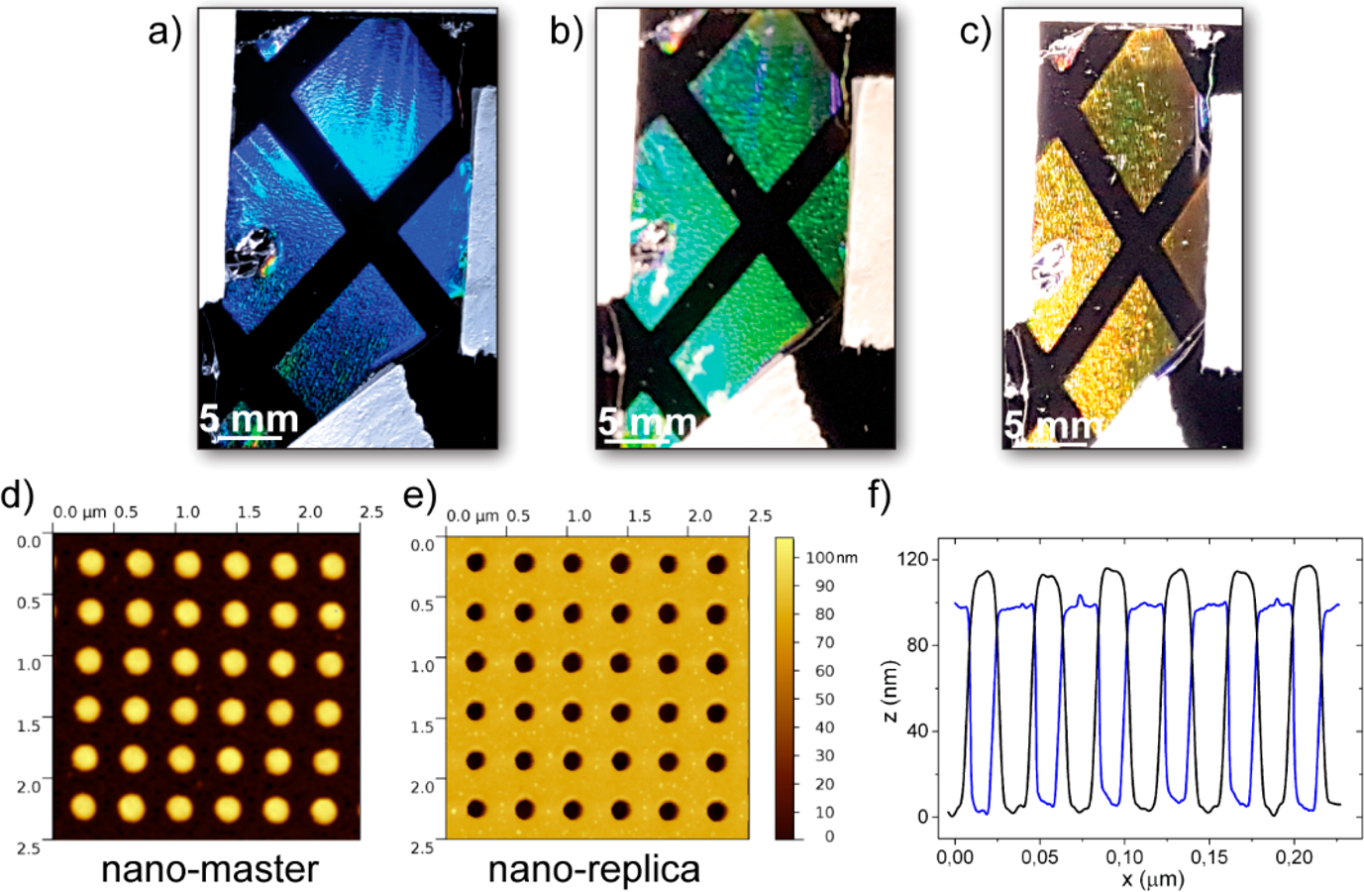
Dielectric colors. (a–c) Photographs of the nanopatterned cellulose film at different angles. (d, e) AFM topography of the polymeric master (d) and the cellulose replica mold (e). (f) Height profiles extracted from the AFM topographies along the indicated lines of the master (black) and the cellulose replica (blue).

The structuring of dielectric films with patterns that have subwavelength periodicities leads to an optical response that is different from diffraction. In this regime the structured dielectric film acts as an effective medium for light^62−66^ and has a nontrivial optical response that leads to the vivid dielectric colors that are observed while rotating the sample with respect to the angle of incidence, as shown in Figure 3a–c. Here the cellulose film was structured by a square array of polymeric nanopillars with 200 nm diameter and a periodicity of 400 nm (Figure 3d) that resulted in a corresponding array of nanoholes as shown in Figure 3e,f. This nanostructured cellulose film acts as a metasurface that diffracts light in the visible range with a strongly wavelength and angle-dependent reflectance, as depicted in Figure S4, which is at the origin of the chromatic effects according to which a pronounced angular color change is provided.

**Figure 4:**
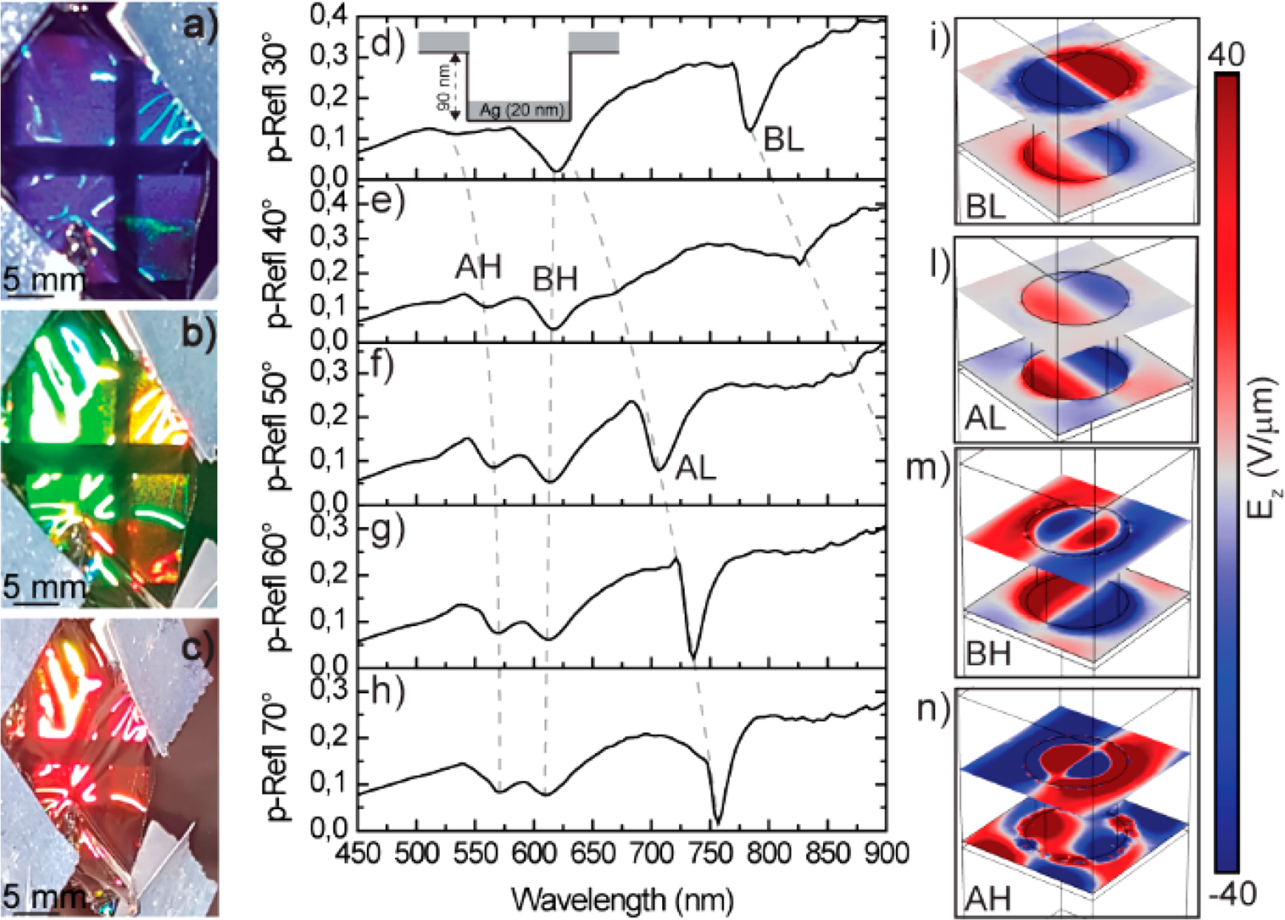
Plasmonic colors. (a–c) Blue, green, and red colors reflected while tilting the plasmonic metasurface, where a thin Ag layer is evaporated on the top of the nanostructured cellulose film. (d–h) Experimentally measured p*-*polarized reflectance of the plasmonic metasurface at (e) 30°, (f) 40°, (g) 50°, (h) 60°, and (i) 70°, showing the presence of four modes consisting of a low-energy bonding (BL) and antibonding (AL) mode and a high-energy bonding (BH) and antibonding (AH) mode. (i–n) Electric field intensity of the four modes simulated *via* COMSOL Multiphysics.

By overcoating the nanostructured cellulose films with metals such as Au or Ag, plasmonic effects can be obtained. In particular, plasmonic colors, where the metalized nanopatterned surface acts as a photonic canvas, have attracted much attention in recent years.^67−70^ Here the plasmonic resonances in the nanostructured metal film lead to maxima in the absorbance of light that depend on the wavelength and angle of incidence. Key requisites for high-quality plasmonic colors are homogeneity of the plasmonic pattern and large area coverage. Both are well fulfilled by the nanostructured and metallized cellulose films, as can be seen by the reflection spectra shown in Figure 4a–c, in which the color is selected by the plasmonic angular dispersion of the metasurface. The deposition of 20 nm of Ag on the nanostructured cellulose film shown in Figure 3e leads to two coplanar and coupled plasmonic square arrays: on the top an array of holes in a thin Ag film, and 90 nm below a corresponding array of metal disks. The plasmonic structure therefore consists of a 2D plasmonic disk–hole matrix as sketched in the inset in Figure 4d. Measurements of the p*-*polarized reflectance at different angles reveal the dispersion of the different plasmonic resonances (Figure 4d–h). Using finite element method (COMSOL) simulations, we can assign the different resonances as low-energy bonding (BL) and antibonding (AL) and high-energy bonding (BH) and antibonding (AH) optical modes, as demonstrated in Figure 4i–n. Details on the plasmonic response leading to such a spectral response are given in Section 5, Figures S5–S8, of the Supporting Information.

The plasmonic resonances in Figure 4d–h should lead to a strong near-field enhancement that can be exploited for surface-enhanced Raman spectroscopy.

**Figure 5:**
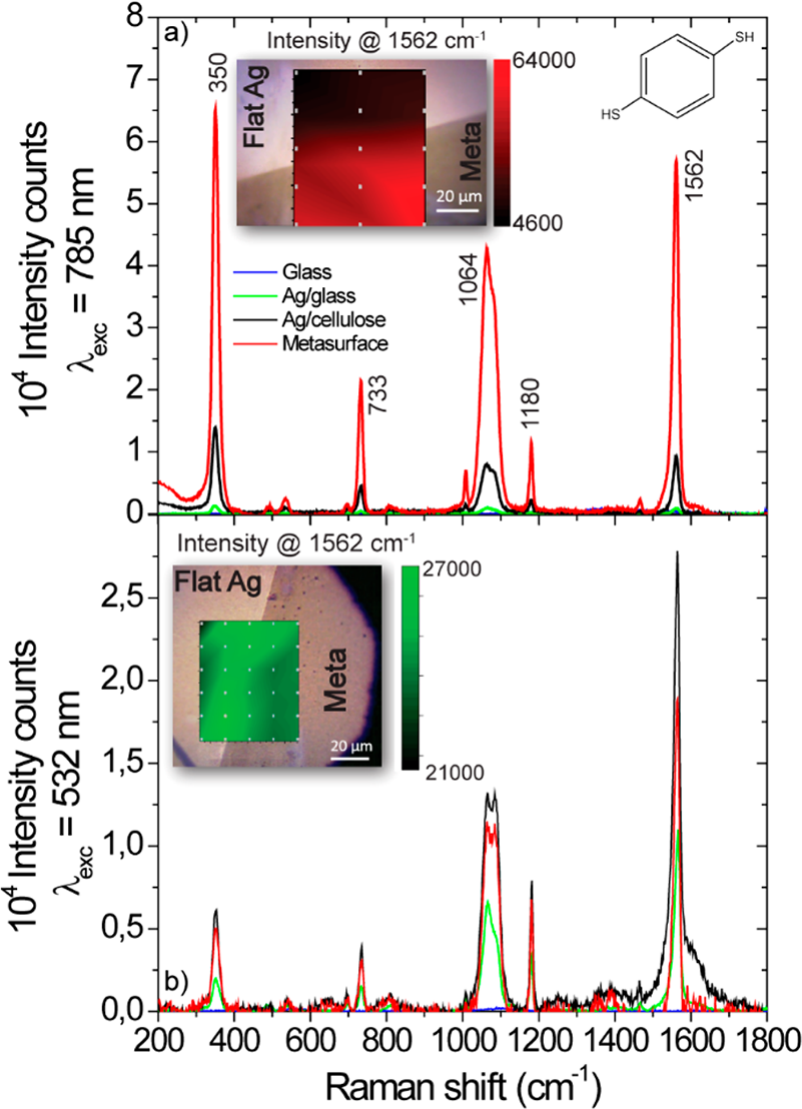
SERS effect. Raman spectrum of 1,4-benzenedithiol acquired on glass (blue), the flat silver (green), Ag-coated cellulose (black), and the nanopatterned (red) area of the sample under excitation at (a) 785 nm and (b) 532 nm. The intensity map in the inset corresponds to the Raman peak at 1562 cm^–1^ of the 1,4-BDT molecules.

For SERS experiments with a microscope setup, the illumination occurs at normal incidence and the signal is collected with an objective with high numerical aperture. Therefore, the resonances in Figure 4d occurring at angles <40° will be the relevant ones, and we can expect a SERS effect at around 620 nm (BH and AL resonances) and 780 nm (BL resonance). The latter resonance matches well with an available laser source in our Raman system, and Figure 5a shows Raman spectra collected under identical conditions with a laser excitation at 785 nm from a layer of 1,4-benzenedithiol molecules on a flat Ag film (black) and from the metallized cellulose metasurface (red). We clearly observe an around 6-fold stronger Raman signal from the metasurface region. Control experiments at a wavelength of 532 nm (Figure 5b, see Section 6 for stability or additional tests), where the metasurface has no plasmonic resonance, show no noticeable SERS enhancement with respect to a planar Ag film, and, therefore, confirm the plasmonic origin of the signal enhancement. The comparison with the Raman signal obtained from bare glass and a Ag film on glass demonstrates that already the metal-coated cellulose film acts as a substrate exhibiting the SERS effect.

## Conclusions

## Methods

### Materials

High-purity microcrystalline cellulose (crystallinity ∼79%) from cotton linters, trifluoroacetic acid, and trifluoroacetic anhydride were purchased from Sigma-Aldrich and used as received. Cocoa pod husks were provided by local vegetable processors as an unusable waste from their production line.

### Cellulose Solution Fabrications

Cellulose and cocoa pod husks were dried in an oven at 40 °C overnight. Cellulose solutions with a final concentration of 15 mg/mL were prepared by dissolving microcrystalline cellulose powder in a mixture TFA/TFAA (2:1, v/v). The complete dissolution took 30 min at room conditions. Similar solutions were prepared with a fine powder of ground cocoa pod husk byproducts. Cocoa pod husk solutions were ready after 3 days at 40 °C with magnetic stirring. Then, the solutions were centrifuged and films were prepared by blending predetermined volumes of cellulose and cocoa wastes in order to obtain a final concentration of 10 wt % of cellulose, *i*.*e*., the minimum amount enough to form a freestanding film after solvent evaporation.

### Fabrication of the Microscale Hexagonal Photonic Crystal and of the Nanoscale Square Lattice Metasurface

**Scheme 2:**
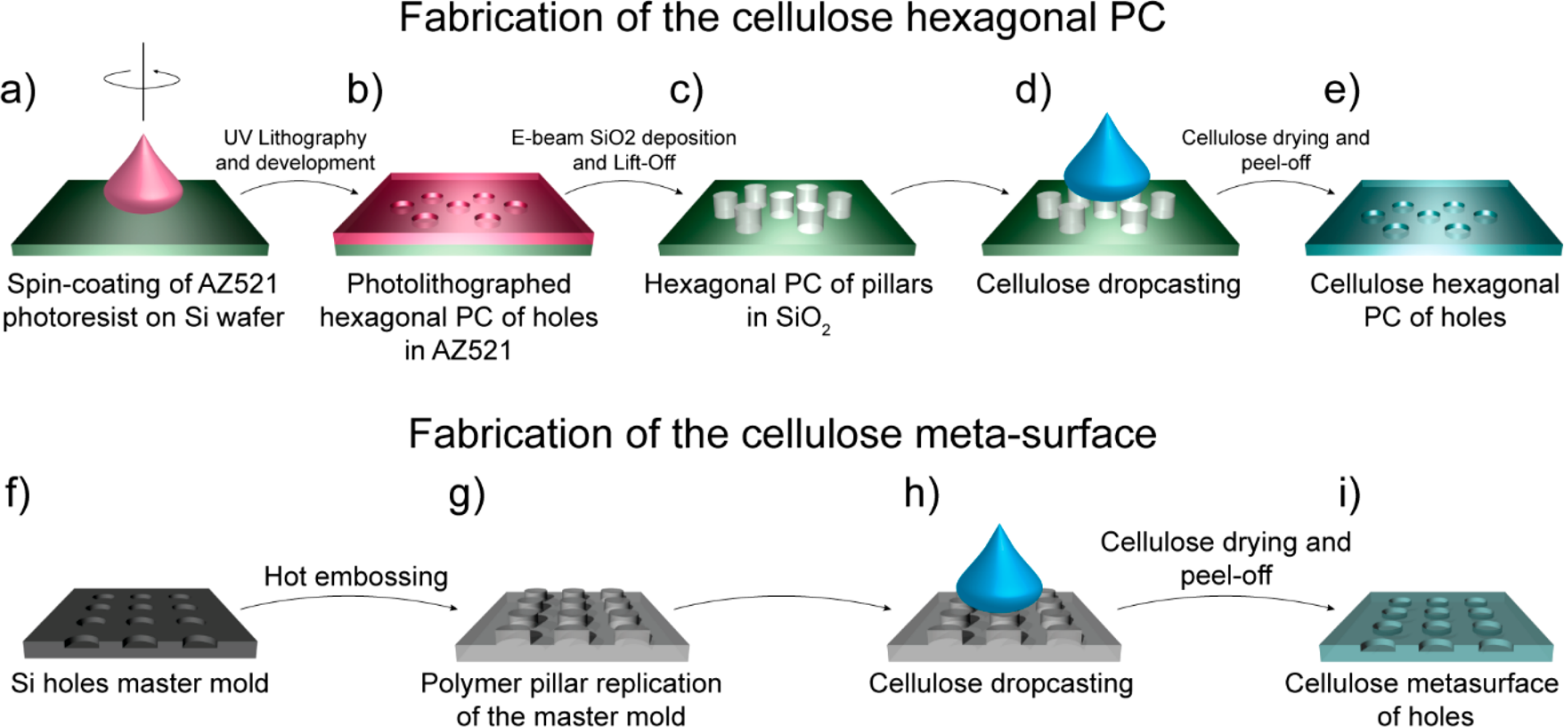
Fabrication of the cellulosed hexagonal PC and of the square-lattice metasurface: (a) Drop-casting of the photoresist (AZ521) on the Si substrate. (b) UV exposure and development to obtain hexagonal PC of holes. (c) Evaporation of 150 nm SiO_2_ by ALD and lift-off to obtain a hexagonal PC of SiO_2_ pillars. (d) Cellulose and cocoa pod husks blend pouring and drying. (e) Peel off of the dried cellulose to obtain the hexagonal PC of cellulose holes. (f) Si master produced by DUV-stepper lithography and reactive ion etching. (g) PMMA replica *via* hot embossing. (h) Cellulose and cocoa pod husks blend pouring on the PMMA master. (i) Peel off of the dried film to obtain the square lattice cellulose metasurface.

The hexagonal photonic crystal master was fabricated *via* a standard photolithography technique as shown in Scheme 2a–e. A standard positive optical resist (AZ 5214 E from MicroChemicals) was spin-coated on a Si wafer and baked at 95 °C for 7 min. The resist was exposed under UV illumination through a 4 cm × 4 cm photomask with the hexagonal photonic crystal pattern for 15 s in hard contact condition. The exposed sample was then developed (developer AZ 726MIF from MicroChemicals) to achieve a hexagonal photonic crystal of holes. A 150 nm thick SiO_2_ layer was then deposited *via* atomic layer deposition (ALD) in a Flexal ALD system from Oxford Instruments. A lift-off process was finally carried out in acetone to remove the underlying resist and leave the hexagonal photonic crystal pattern made of 150 nm SiO_2_ cylinders. A cellulose solution was then poured on the SiO_2_ master, ensuring its complete and homogeneous coverage. The film was dried under a chemical hood until complete solvent evaporation. After that, cellulose and cocoa pod husk films were carefully stripped from the masters. Finally, the films were dried under vacuum at room temperature for 24 h to remove any solvent residue and stored at 44% RH for 7 days before analysis to ensure the reproducibility of the measurements.

The nanoscale square lattice metasurface was fabricated *via* the classic replica-molding technique as shown in Scheme 2f–i. A master mold was prepared *via* deep-UV-stepper lithography and reactive ion etching in a silicon wafer, described elsewhere.^67,71^ Starting from a master mold of a nanohole matrix, a polymeric (Ormocomp, Micro Resist Technology GmbH) square lattice metasurface was replicated *via* hot embossing. The resulting metasurface was used as a stamp for all replications. Cellulose was then poured on top of the polymeric metasurface, allowed to dry, and peeled off as described before to obtain a square lattice metasurface of holes.

### Spectroscopic Ellipsometry

Spectroscopic ellipsometry (V-VASE ellipsometer, Woollam) was used to determine the refractive index and transmission spectrum of the obtained cellulose film. This investigation allowed retrieving the optical parameters of the cellulose film. Details on the fitting procedure are provided in Section 1 and Figure S1 of the Supporting Information.

### Vapor Deposition of 1,4-Benzenedithiol Molecules and Micro-Raman Spectroscopic Characterization

The target molecule, 1,4-benzenedithiol (1,4-BDT, 97%, Alfa Aesar), was placed on the cellulose substrates by vapor deposition. The deposition was carried out in a closed vial (20 mL) placing 1 mg of 1,4-BDT on the bottom and the cellulose substrate (with both surfaces, flat silver and nanostructures) supported on the top. By applying a mild heating on the bottom (hot plate at 80 °C), the 1,4-BDT partially sublimated (clearly seen at the vial walls as white “fog” (fume)), creating a homogeneous coverage of the substrate. Raman spectra of the 1,4-BDT molecules deposited on the substrate were acquired using a Renishaw Via micro-Raman microscope equipped with a 50× (0.75 NA) objective using two excitation wavelengths, 532 and 785 nm, and an incident power of less than 1 mW to avoid damaging the samples during the measurement. Raman maps (*ca*. 80 × 80 μm^2^) of the 1,4-BDT spectrum in the sample at the bordering flat-silver/nanostructured were recorded with a step size in *x* and *y* direction in the 20–30 μm range. To obtain a better signal-to-noise ratio, the Raman spectra and maps in the main text were acquired using 1 accumulation of 10 s in all the cases. The stability experiments reported in Section 6 of the SI have been carried out with a WITec Alpha 300 microscope. The color Raman intensity maps shown in the main text and Section 6 of the SI are the result of the spatial interpolation of the Raman intensity at the band 1562 cm^–1^ from the spectra collected of the 1,4-BDT molecules at the points displayed as gray squares in each panel. Control experiments to evaluate the Raman intensity standard deviation inside whole flat-silver and nanostructured areas were done by collecting Raman maps, keeping the same trend observed at the borders and confirming the results.

### Biodegradation Tests

Biodegradability was evaluated in both seawater and soil. Biodegradability in seawater was tested through a standard biochemical oxygen demand (BOD) test by measuring of the oxygen amount consumed during a biodegradation reaction in water.^72^ For each sample, three measurements were collected and the results were averaged to obtain a mean value. Carefully weighed samples (∼200 mg) were finely minced and immersed in 432 mL bottles containing seawater collected from the Genoa (Italy) area shoreline. Oxygen consumed during the biodegradation process was recorded at different time intervals by using sealed OxyTop caps on each bottle that can assess the oxygen levels. BOD from blank bottles filled with only seawater was also measured for reference.

For the biodegradation in soil, the test was carried out following the standard ISO 17556. Briefly, ∼100 mg samples were cut in 4 mm × 4 mm pieces and mixed with 100 g of selected soil collected from a noncommercial forest area from San Sebastian (Spain). The soil was taken from the surface layer and sieved in order to remove particles >2 mm size. Plant materials, stones, and other inert materials were also removed. Then, the mixtures were introduced in 1 L glass reactors and kept at 25 °C with a constant flow of CO_2_ free air of 100 mL/min. The amount of carbon dioxide evolved during the biodegradation process was monitored during six months. Degradability from reactors containing just soil was used as reference. For each sample, three measurements were run in parallel, and the results were averaged to obtain a mean value. The level of biodegradation expressed in percent was determined by comparing the net carbon dioxide evolved with the theoretical amount (amount expected in the case of total oxidation of the test material), as indicated by the following equation:
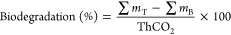
where ∑*m*_*T*_ is the amount of carbon dioxide, in milligrams, evolved in the test flask between the start of the test and the time being considered, ∑*m*_B_ is the average, considering the 3 replicas, amount of carbon dioxide, in milligrams, evolved in the blank flask between the start of the test and the time being considered, and ThCO_*2*_ is the theoretical amount of carbon dioxide in milligrams evolved by the test material.

## Supporting Information Available

Ellipsometry and transparency experiments; evaluation of the diffraction efficiency; SEM analysis; reflectance spectra of the nanostructured cellulose samples; COMSOL simulation of the plasmonic response of the metalized cellulose metasurface; additional Raman analysis (PDF)
