# Supplementary material for: Biodegradable and Insoluble Cellulose Photonic Crystals and Metasurfaces
Source: ACS Nano. 2020 Jun 19;14(8):9502–11. doi: 10.1021/acsnano.0c03224 (PMC8008445; doi:10.1021/acsnano.0c03224)
Supplement: Supplementary file 1 — nn0c03224_si_001.pdf [file nn0c03224_si_001.pdf]

SUPPORTING INFORMATION FOR

Biodegradable and Insoluble Cellulose Photonic Crystals and Metasurfaces

Vincenzo Caligiuri[ʎ†ʕ], Giacomo Tedeschi[†], Milan Palei[†◊], Mario Miscuglio[※], Beatriz Martin-Garcia[†ϒ], Susana Guzman-Puyol[•†], Mehdi Keshavarz Hedayati[¥], Anders Kristensen[□], Athanassia Athanassiou[†], Roberto Cingolani[†], Volker J. Sorger[※], Marco Salerno[‡], Francesco Bonaccorso[†,a], Roman Krahne[†] and José Alejandro Heredia-Guerrero*[•†]*

[†]Istituto Italiano di Tecnologia, Via Morego 30, 16163 Genova, Italy

[ʎ]Dipartimento di Fisica, Università della Calabria, 87036 Rende, Italy

[ʕ] CNR Nanotec, Università della Calabria, 87036 Rende, Italy

[ϒ]CIC nanoGUNE, Tolosa Hiribidea 76, 20018 Donostia-San Sebastian, Basque Country, Spain.

[◊] Department of Electrical Engineering, University of Notre Dame, IN,46556, USA

[•]IHSM La Mayora, Departamento de Mejora Genética y Biotecnología, Consejo Superior de Investigaciones Científicas, E-29750 Algarrobo-Costa, Málaga, Spain.

[※]Department of Electrical and Computer Engineering, George Washington University, Washington, DC 20052, USA

[¥]Department of Engineering, Durham University, Durham DH1 3LE, United Kingdom

[□]Department of Health Technology, Technical University of Denmark, DK-2800 Kongens Lyngby, Denmark

[‡]Materials Characterization Facility, Istituto Italiano di Tecnologia, Via Morego 30, 16163 Genova, Italy

[a]BeDimensional Srl., Via Albisola 121, 16163 Genova, Italy

* CORRESPONDING AUTHORS: ja.heredia@csic.es, vincenzo.caligiuri@unical.it

Keywords: cellulose, cocoa agro-waste, biodegradability, water insolubility photonic crystals, meta-structures, plasmonic colours, SERS.

Section 1 - Ellipsometry and transparency experiments:

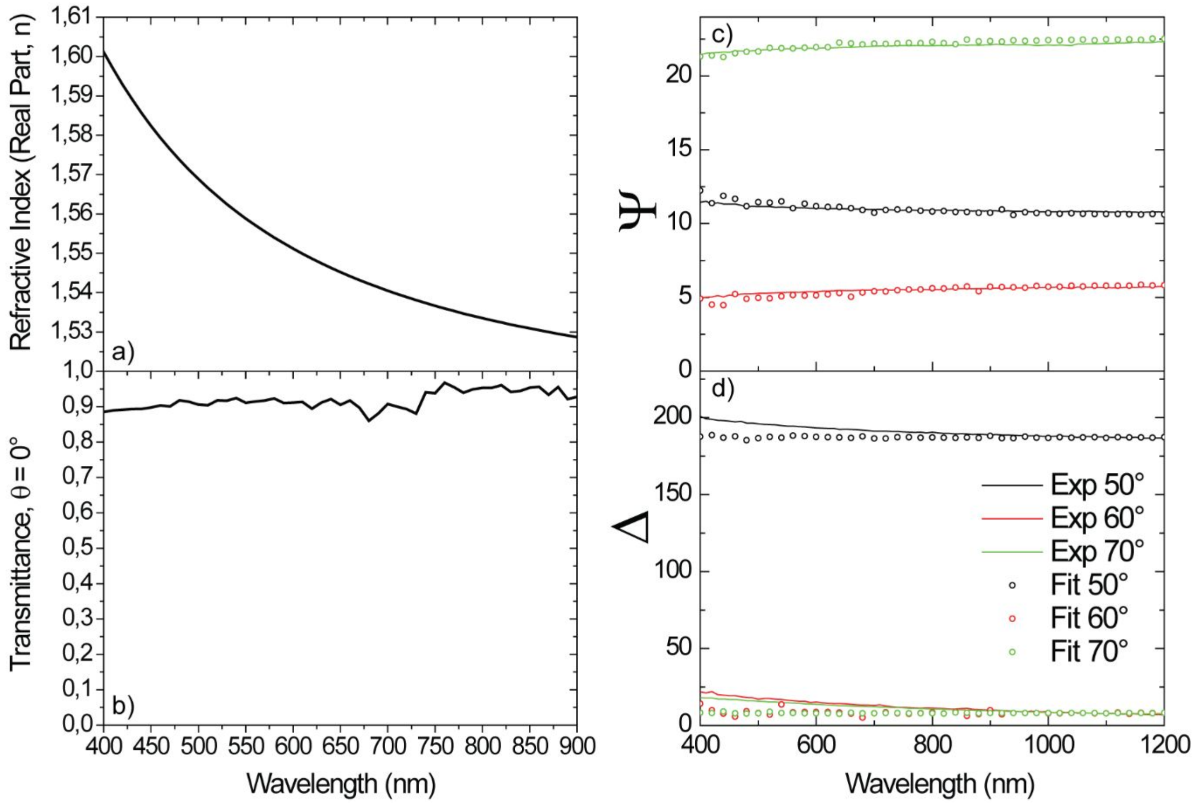

Figure S1 - Refractive Index and Transmittance. Refractive index (a) and transmittance (b) of the fabricated cellulose layer.

Figure S1a shows the real part of the refractive index of the cellulose, calculated by fitting the ellipsometrical angles ψ and Δ shown in **Figure S1c,d.** The measured refractive index is in line with the one of the most common polymers used in photonic applications. **Figure S1b** shown the transmittance of the cellulose layer that is around 90% all over the VIS-NIR range.

Section 2 – Evaluation of the diffraction efficiency

The diffraction efficiency of a system is expressed as:

$\eta = P_d / P_{in}$

Where P_d is the diffracted power and P_{in} is the incident one. Thanks to its transparency, the diffraction efficiency for the hexagonal diffraction grating made of pure cellulose can be evaluated by simply following the procedure indicated in **Figure S2a**. As a first step, the incident power is acquired *via* a photodiode connected to an oscilloscope. In the second step, the power of the zeroth order transmitted beam is acquired. The difference in power between these two values is directed to all the diffraction orders shown in **Figure S2c**. In the case of the pure cellulose grating, a diffraction efficiency of about 20% is detected, in line with the typical performances of hexagonal diffraction gratings. In the case of the low-transmittance cocoa blend it is not possible to use this technique and the diffraction efficiency has to be evaluated by measuring the intensity of each single diffracted beam, as illustrated in the sketch of **Figure S2b**. **Figure S2d** shows that the power diffracted into orders higher than the second is negligible, therefore the diffraction efficiency can be evaluated by measuring the intensity of the 1st and 2nd order diffracted beams. In this case, the diffraction efficiency is close to the 14 %.

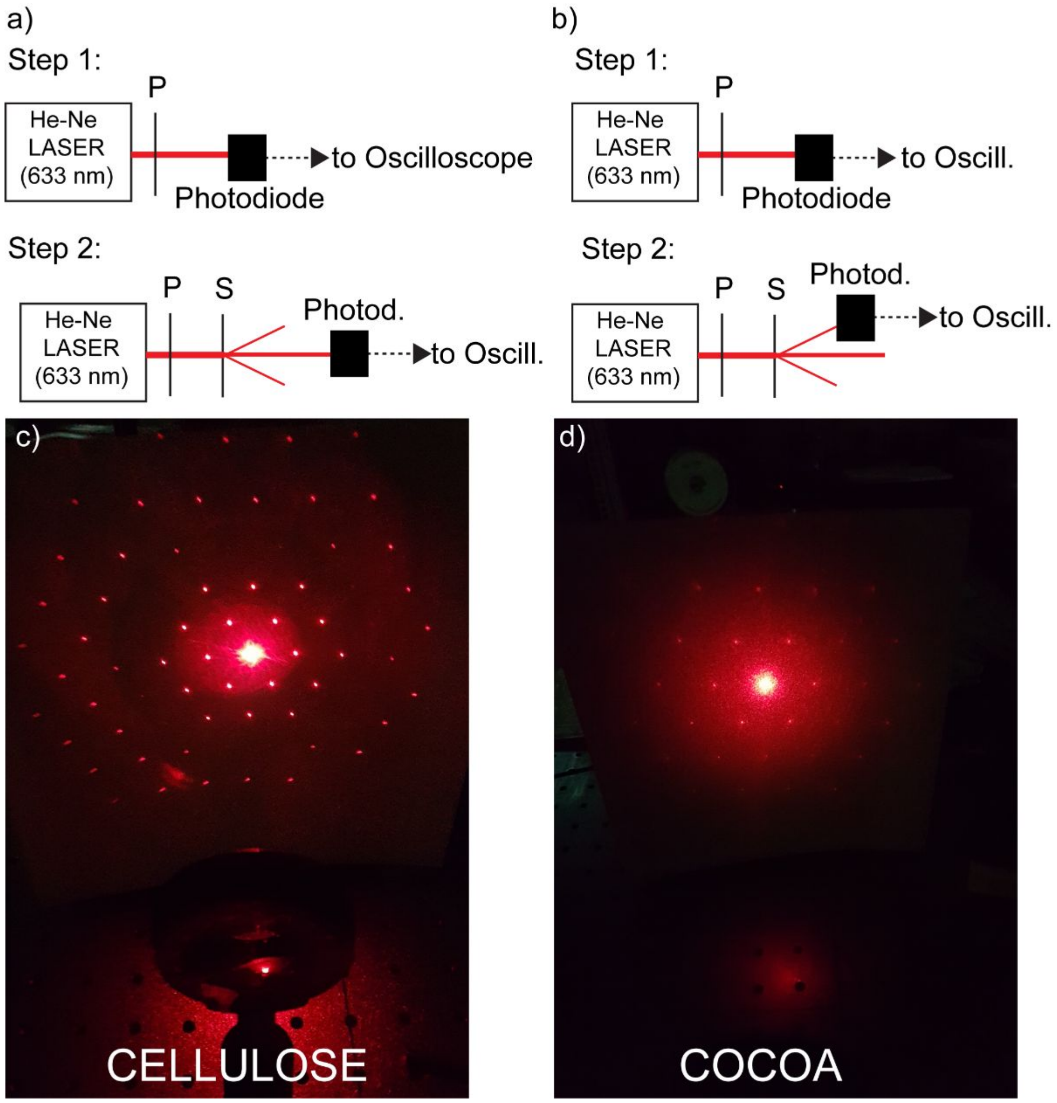

Figure S2 - Diffraction efficiency measurements. In the case of the pure cellulose hexagonal diffraction grating, the diffraction efficiency can be measured by simply using the technique shown in panel (a). Thanks to its high transparency, a significant number of diffracted order is visible, as shown in (c). In the case of the cocoa diffraction grating, the low transparency requires the setup shown in (b). The diffraction pattern is shown in (d).

Section 3 – Scanning Electron Micrograph analysis

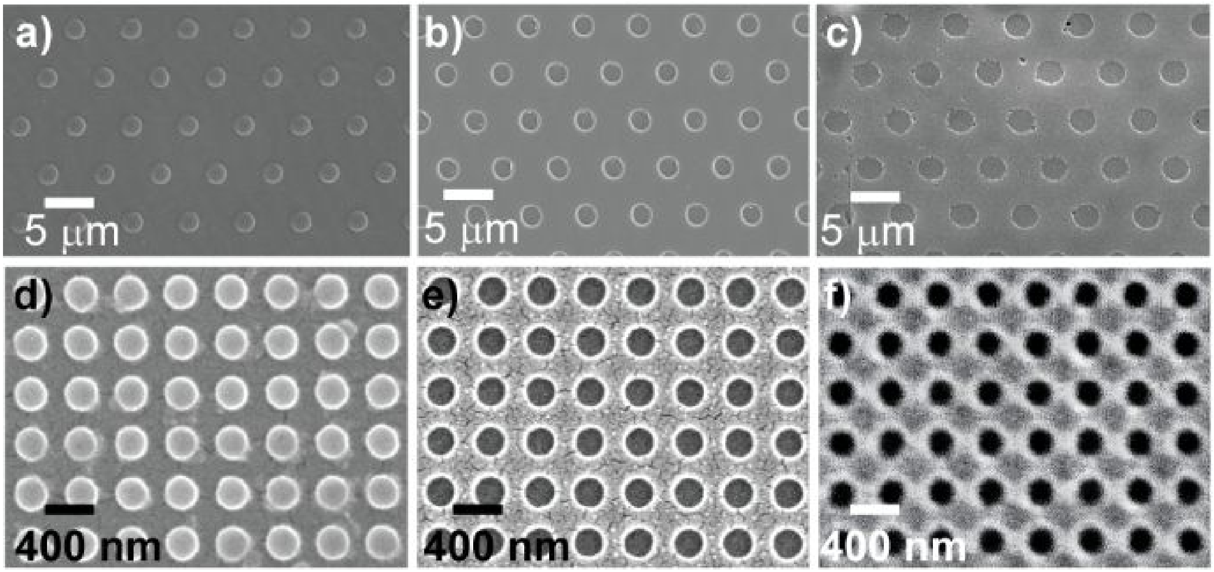

Figure S3 – Scanning Electron Microscopy images. SEM images of the (a) micro- and (d) nano-scale masters compared respectively to the (b,c) micro- and (e,f) nano-scale replica in (b,e) pure cellulose and (c,f) cocoa.

Section 4 – Reflectance spectra of the nanostructured cellulose samples

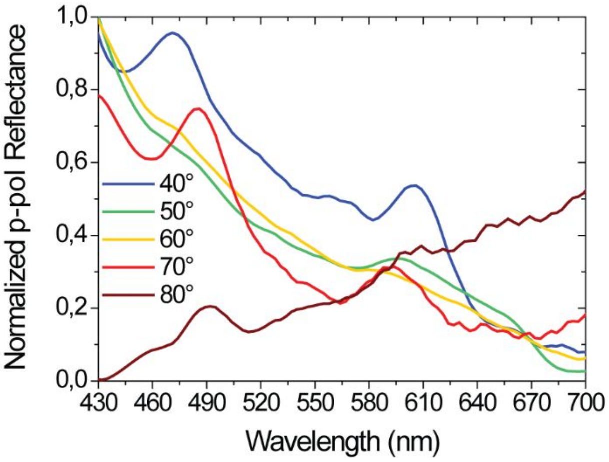

Figure S4 – Nanostructured Cellulose reflectance. P-Polarized Reflectance spectrum of the cellulose metasurface.

P-Polarization reflectance spectra of the cellulose metasurface. The peculiar reflection response in Figure S4 is responsible for the chromatic effect shown in Figure 3a,c of the main manuscript.

Section 5 – COMSOL simulation of the plasmonic response of the metalized cellulose metasurface

Here we discuss the optical response of the periodic nano-structures and correlate the simulation results to our experimental measurements. The simulated structure corresponds to a periodic array (L=400nm) of holes

(diameter = 200nm) in a cellulose film (90 nm thick). The cellulose film is covered with a 20 nm thick layer of silver. (**Figure S5**) The refractive index of silver (Ag) is taken from Johnson and Christy (1972: n,k 0.188-1.937 μm), while the dielectric constant of the cellulose film is set at $n_{cellulose}$ = 1.5.

The numerical simulation solves Maxwell's equation with periodic Floquet's boundary conditions at the side of the unit cell. To simulate open boundaries at the top and the bottom of the nanostructured array, two perfectly matched layers are added, in correspondence of the excitation and collection port, to avoid unphysical reflections. Convergence mesh analysis was applied to reach stationary results (minimum mesh quality 0.7).

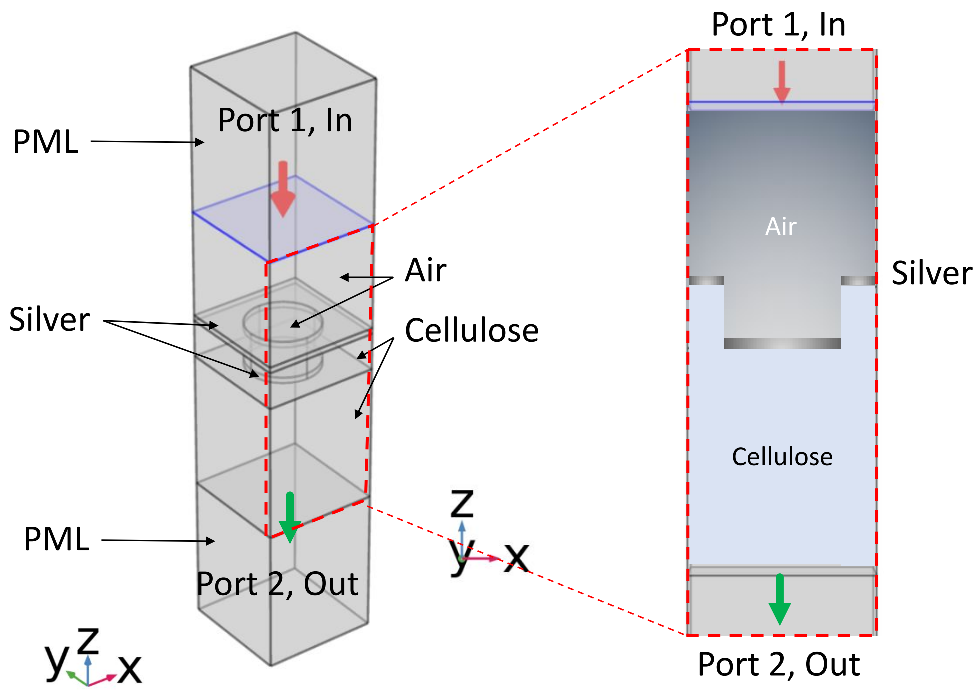

Figure S5 - Structure considered in the numerical studies.

A full-wave 3-dimensional simulation is used for investigating the transmittance spectrum of the array under the condition of normal incident plane wave and, at the resonant peaks, the corresponding near-field profile, Re(Ez), the real part of the vertical component of the electric field. In **Figure S6** we show the transmission spectra for the structure reported in **Figure S5** by means of both FDTD (Lumerical) and finite elements method (Comsol Multiphysics). The array is illuminated by a plane wave linearly polarized along x-axis of the structure at normal incidence. The spectral response is simulated from VIS to NIR (400 nm to 1.2 μm) with a resolution

that allows to resolve the sharpest spectral features (1 THz). The simulated transmission spectra are in excellent agreement with the experiment in terms of spectral position. The difference in depth and width of the dips in the transmittance spectrum between the measurements and the numerical result is mainly due to the finite size of the array in the experimental case. Other minor shifts and broadening of the peaks can be ascribed to the imperfections in both size and periodicity of the array in the experiments.

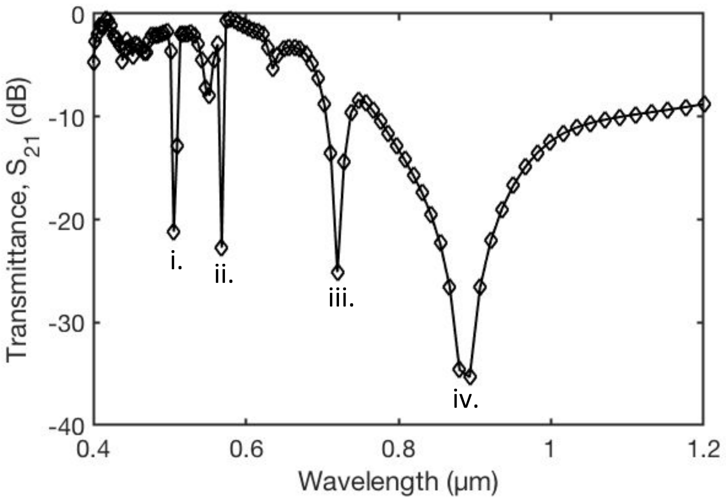

Figure S6 - Optical response of the 2-dimensional periodic array. Transmittance (S21) reported in dB. The dips in the transmittance spectrum represent the resonant modes of the nano-structured array: Low-Energy Bonding (BL, iv) and Antibonding (AL, iii), and their overtones, namely High-Energy Bonding (BH, ii) and Antibonding (AH, i).

The electric field distribution at the lower energies (longer wavelengths) show the fundamental plasmon hybridization modes due to disk-hole coupling, namely bonding (880 nm) and antibonding (760 nm). Higher energy modes correspond to the second harmonics of the bonding (570) and antibonding (507) modes. The electric field distribution for the lower energy modes (**Figure S7** iii-iv) shows the dipolar nature of the resonances of the hole and the disk, in counter-phase for the bonding mode and in phase for the antibonding mode. At higher energy instead quadrupole-like modes are either in-phase (**Figure S7** i) or out-of-phase (**Figure S7** ii).

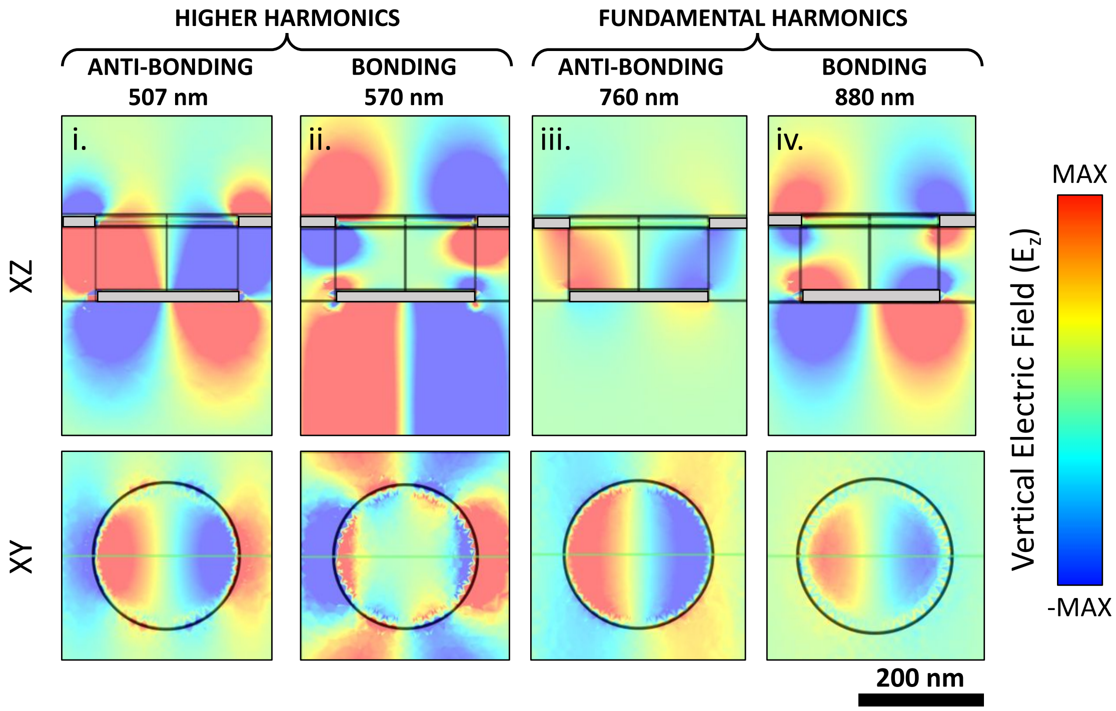

Figure S7 - Numerical study of the plasmonic modes of the 2-dimensional grating. Simulated spatial distribution of the vertical component of the electric field (Ez) of the first 4 modes (i-iv) in the xz and xy plane for cylindrical holes (height 120 nm, radius 125 nm) in a cellulose film (nCellulose ~ 1.5) covered with a thin (20 nm) silver film. An electromagnetic wave is impinging orthogonally to the structure with a polarization parallel to the x-axis. Field distribution of the Low-Energy Bonding (BL) (iv), its second harmonic (BH) (ii) and the antibonding mode (AL) (iii) and its higher energy overtone (AH) (i).

Figure S8 shows the energy diagram illustrating the hybridization of the coupled plasmonic modes of the disks and holes of the structure into a low energy mode (bonding, BL) and a high energy mode (antibonding, AL). The hybridization of the higher order plasmonic modes of the disk and the hole corresponds to the hybridization overtones (AH, BH).

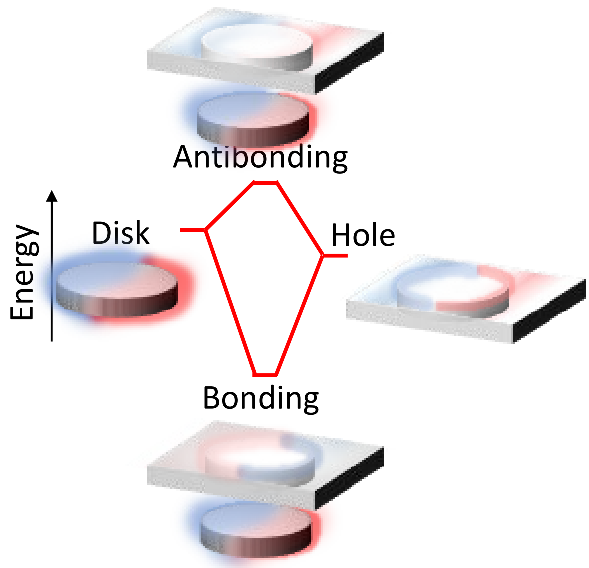

Figure S8 Plasmonic hybridization due to disk-hole coupling. The dipole modes of the hole and the disk hybridize forming a symmetric (antibonding) mode with higher resonance frequency, and anti-symmetric (bonding) mode, characterized by a lower resonance frequency (longer wavelength).

Section 6 – Additional Raman analysis

We carried out tests to evaluate the Raman spectrum of the 1,4-benzenedithiol molecule (1,4-BDT) and possible substrate damaging induced by laser exposure to establish the experimental conditions for the Raman measurements. Using 532 nm laser, we acquired the 1,4-BDT Raman spectrum with 1 accumulation (1 s acquisition), 10 accumulations (1 s acquisition each one) and 1 accumulation (10 s acquisition) in the Ag-coated cellulose substrate at the nanostructured regions in different points. As shown in the **Figure S9a**, there are no differences in the spectral shape or additional features to the ones ascribed to BDT molecule, only a better signal-to-noise ratio, passing from 3926 counts (1s) to 30360 (10 accumulations of 1s) and 28784 (10s), thus as expected □10 Raman intensity enhancement, indicating that there are no exposure-induced changes in the deposited 1,4-BDT molecule or the substrate. Additionally, we performed stability tests by successive Raman acquisitions of 1s in an area □100x100 μm^2 with 15 repetitions (cycles) with a time-gap of 1s and 532 nm laser. As can be seen in Figure S9b-c, the statistics done in 15 points and the corresponding Raman intensity maps demonstrate a clear retention of the Raman intensity at the Raman peak 1562 cm^{-1}.

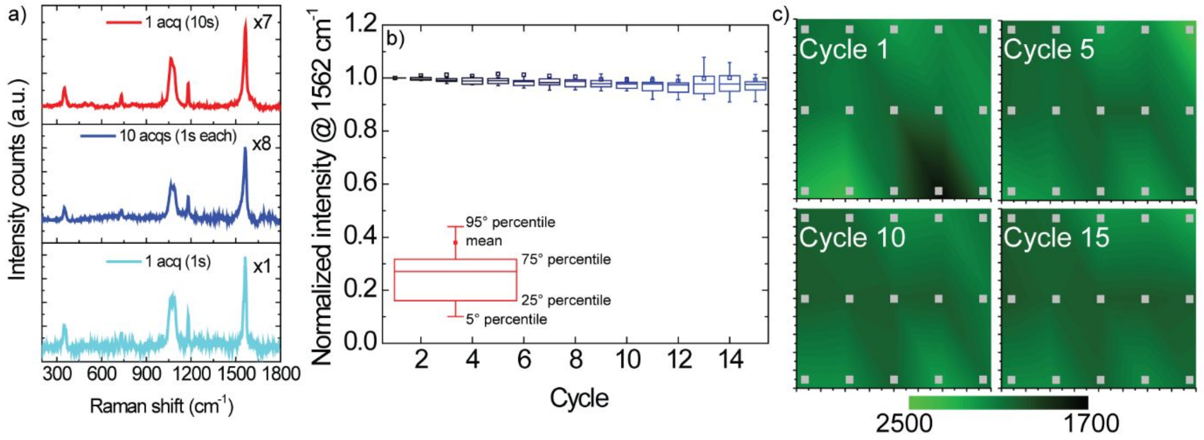

Figure S9. (a) Raman spectrum of 1,4-benzenedithiol acquired on the metallized nano-patterned cellulose area of the sample under excitation at 532 nm, using different acquisition conditions: 1 accumulation -acq- (1 s acquisition) (cyan), 10 accumulations (1 s acquisition each one) (blue) and 1 accumulation (10 s acquisition) (red). (b) Statistical analysis of the stability cycles carried out with 15 consecutive acquisitions (1s exposure and 1s waiting) on the metallized nano-patterned cellulose area of the sample under excitation at 532 nm. The corresponding intensity map of the Raman peak at 1562 cm^{-1} of the 1,4-BDT molecules at cycles 1st, 5th, 10th and 15th, are also shown.
